# Supplementary material for: Characterization of Engineering Plastics Plasticized Using Supercritical CO2
Source: Polymers (Basel). 2020 Jan 6;12(1):134. doi: 10.3390/polym12010134 (PMC7022295; doi:10.3390/polym12010134)
Supplement: Supplementary file 1 [file polymers-12-00134-s001.pdf]

polymers

MDPI

Supplementary Materials

Characterization of Engineering Plastics Plasticized Using Supercritical CO_2

Masaki Watanabe, Yoshihide Hashimoto, Tsuyoshi Kimura and Akio Kishida *

Department of Material-Based Medical Engineering, Institute of Biomaterials and Bioengineering, Tokyo Medical and Dental University, Tokyo 101-0062, Japan; masaki.wm.watanabe@jp.ricoh.com (M.W.); hashimoto.atrm@tmd.ac.jp (Y.H.); kimurat.mbme@tmd.ac.jp (T.K.)

* Correspondence: kishida.mbme@tmd.ac.jp; Tel.: +81-3-5280-8028

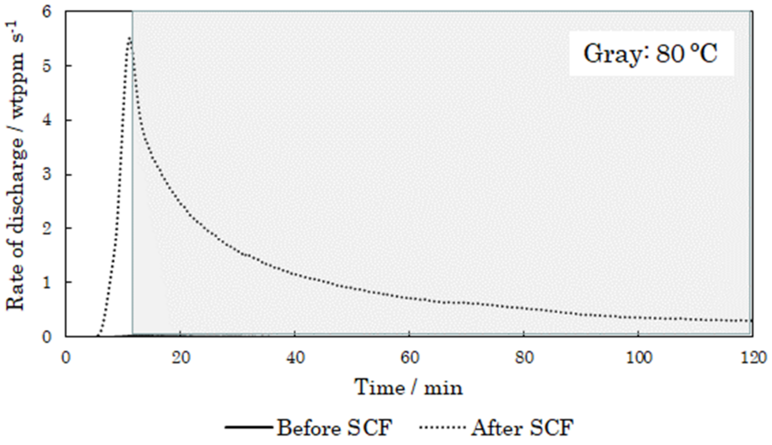

Figure S1. Amount of CO_2 used in plasticizing PLLA. The amount of CO_2 in PLLA was analyzed using Temperature Programmed Desorption-Mass Spectrometry (TPD–MS). The amount of CO_2 released upon heating using a heater at 80 °C (Small-8, TRC Co., Ltd., Tokyo, Japan) was analyzed using MS (GC/MS QP2010(9), Shimazu Co., Ltd., Kyoto, Japan). CO_2 was released upon melting PLLA (after supercritical treatment), but small amounts of CO_2 continued to be released even after 100 min. Untreated PLLA (before supercritical treatment) did not release CO_2. As mentioned in Table 2, the amounts of CO_2 released in experiments 1 (slight melting) and 3 (enough melting) were 360 wt ppm and 7500 wt ppm, respectively.
